# FDA grants approval to the RSV vaccine (nirsevimab-alip) for all infants: a leap forward for shielding the smallest

**DOI:** 10.1097/JS9.0000000000000698

**Published:** 2023-09-02

**Authors:** Abubakar Nazir, Rida Fatima, Awais Nazir

**Affiliations:** a Oli Health Magazine Organization, Research, and Education, Kigali, Rwanda; b Department of Medicine, King Edward Medical University, Lahore, Pakistan

Highlights

- Respiratory syncytial virus (RSV) is a ubiquitous pathogen that affects the respiratory tract, primarily targeting infants, young children, and the elderly. It is one of the leading causes of severe lower respiratory tract infections in infants.
- Recently, the United States Food and Drug Administration (FDA) approved the RSV injection for all infants. The approval of Beyfortus relied partly on data obtained from the phase 3 MELODY trial.
- It is found that a single injection of nirsevimab administered before the RSV season protected healthy late-preterm and term infants from medically attended RSV-associated lower respiratory tract infection.

Respiratory syncytial virus (RSV) is a ubiquitous pathogen that affects the respiratory tract, primarily targeting infants, young children, and the elderly. It is one of the leading causes of severe lower respiratory tract infections in infants and can pose significant health risks, especially to premature babies and those with underlying health conditions. RSV is highly contagious and spreads through respiratory droplets, making it a major concern for healthcare^1^. RSV has long been a cause of concern for parents and caregivers due to its potential to lead to severe respiratory illness in infants. In the United States, approximately 80 000 children under the age of 5 are hospitalized each year due to RSV infection. While most cases are mild, infants under 6 months, premature infants, and children with weakened immune systems or neuromuscular disorders are particularly susceptible to severe illness, according to the Centers for Disease Control and Prevention^1^. Previously, there were no licensed vaccines specifically for RSV available for routine use in children^1^. However, a ray of hope has emerged in the realm of pediatric healthcare with the recent approval of the RSV injection by the United States Food and Drug Administration (FDA) for all infants. The monoclonal antibody Beyfortus (nirsevimab-alip), which has already received approval for use in Europe and Canada, is intended for administration to newborns and infants who are either born during or entering their first RSV season. Additionally, it is recommended for children up to 24 months old who face a heightened risk of severe RSV infection during their second RSV season^2^.

The approval of Beyfortus relied partly on data obtained from the phase 3 MELODY trial^2^. The trial demonstrated that the injection led to a 74.9% reduction in medically attended lower respiratory tract infections related to RSV compared to the placebo group [with a 95% confidence interval (CI) of 50.6–87.3; *P*<0.001]^3^. A total of 1490 infants were included in the study, with 994 infants assigned to the nirsevimab group and 496 infants to the placebo group. Medically attended RSV-associated lower respiratory tract infection was observed in 12 infants (1.2%) in the nirsevimab group and 25 infants (5.0%) in the placebo group. This indicates that nirsevimab demonstrated an efficacy of 74.5% [95% confidence interval (CI), 49.6–87.1; *P*<0.001] in preventing RSV-associated lower respiratory tract infection^3^. Regarding hospitalization due to RSV-associated lower respiratory tract infection, 6 infants (0.6%) in the nirsevimab group and 8 infants (1.6%) in the placebo group required hospitalization. The efficacy of nirsevimab in reducing hospitalizations was estimated at 62.1% (95% CI, −8.6 to 86.8; *P*=0.07). Thus, it was found that a single injection of nirsevimab administered before the RSV season protected healthy late-preterm and term infants from medically attended RSV-associated lower respiratory tract infection^4^.

Widespread RSV vaccination can have a significant positive impact on public health, particularly in reducing the burden of RSV infections in infants. By vaccinating infants against RSV, the number of severe RSV-related lower respiratory tract infections could significantly decrease, resulting in a reduction in hospitalizations and associated healthcare costs. Widespread vaccination may not only protect vaccinated infants but also provide indirect protection to unvaccinated individuals within the community, including other vulnerable populations, by reducing overall RSV transmission rates^4,5^. By preventing RSV-related hospitalizations and medical interventions, widespread vaccination can lead to significant economic savings for healthcare systems and families alike. While exceptionally rare, some concerns have been raised about vaccine-related encephalitis. However, extensive safety monitoring during clinical trials and post-approval surveillance has not shown any evidence of a causal link between RSV vaccination and encephalitis^5^. The RSV vaccine is recommended for all infants born prematurely, before 29 weeks of gestation, and infants with chronic lung disease. Additionally, infants born at 29–35 weeks of gestation may also benefit from the vaccine during RSV season, typically in the colder months. Healthcare providers usually administer the RSV injection in multiple doses during the RSV season. It is crucial for caregivers to strictly adhere to the recommended schedule to ensure optimal protection for their infants. Caregivers should consult their pediatrician or healthcare provider to assess their infant’s eligibility for the RSV vaccine^5^.

## Ethical approval

Ethics approval was not required for this article.

## Consent

Informed consent was not required for this article.

## Sources of funding

Not applicable.

## Author contribution

A.N., R.F., and A.N.: conceptualization of ideas, critical reviews with comments, and final draft. All authors approved the final manuscript.

## Conflicts of interest disclosure

There are no conflicts of interest.

## Research registration unique identifying number (UIN)

Not applicable.

## Guarantor

Abubakar Nazir, Oli Health Magazine Organization, Research and Education, Kigali, Rwanda; E-mail: abu07909@gmail.com; ORCID ID: 0000-0002-6650-6982.

## Data availability statement

Not applicable.

## Provenance and peer review

Not commissioned, externally peer-reviewed.

## References

[1] Borchers AT Chang C Gershwin ME. Respiratory syncytial virus – a comprehensive review. Clin Rev Allergy Immunol 2013;45:331–379. 23575961 10.1007/s12016-013-8368-9 PMC7090643

[2] Gillespie L. RSV injection approved by FDA for all infants. Medscape, 2023. Accessed 7 August 2023. https://www.medscape.com/viewarticle/994444

[3] Wilkins D Yuan Y Chang Y. Durability of neutralizing RSV antibodies following nirsevimab administration and elicitation of the natural immune response to RSV infection in infants. Nat Med 2023;29:1172–1179. 37095249 10.1038/s41591-023-02316-5 PMC10202809

[4] Hammitt LL Dagan R Yuan Y. Nirsevimab for prevention of RSV in healthy late-preterm and term infants. N Engl J Med 2022;386:837–846. 35235726 10.1056/NEJMoa2110275

[5] Karron RA Atwell JE McFarland EJ. Live-attenuated vaccines prevent respiratory syncytial virus-associated illness in young children. Am J Respir Crit Care Med 2021;203:594–603. 32871092 10.1164/rccm.202005-1660OC PMC7924568

